# Impact of SARS-CoV-2 pandemic on mental health in the elderly: perspective from a psychogeriatric clinic at a tertiary hospital in São Paulo, Brazil

**DOI:** 10.1017/S1041610220001180

**Published:** 2020-06-11

**Authors:** Orestes V. Forlenza, Florindo Stella

**Affiliations:** Laboratory of Neuroscience (LIM-27), Department and Institute of Psychiatry, Hospital das Clínicas, Faculdade de Medicina da Universidade de São Paulo (HCFMUSP), São Paulo, Brazil

## Overview of coronavirus disease 2019 pandemic in Brazil

Recent studies emphasize the high frequency of subsyndromal mental abnormalities in the general population during the new coronavirus pandemic, affecting up to one-third of the exposed individuals (Rajkumar, 2020). These manifestations can be represented by a simple increase in the self-perception of stress, but also by a higher prevalence of anxiety and depression symptoms, sleep complaints and relapse of pre-existing psychiatric disorders (Pfefferbaum and North, 2020). This perspective has been depicted by early population studies conducted in eastern Asia in the first months of the pandemic but seems to be replicable in Western countries as well. In Brazil, much attention is being paid to this secondary but not less important wave of morbidity. The rapidly progressive dissemination of the viral infection causing a consistent growth of incidence rates, along with the high clinical severity of a substantial proportion of coronavirus disease 2019 (COVID-19) cases, may warrant the outburst of a true mental health crisis in the general population. To date, scientific publications addressing mental health issues in the Brazilian COVID-19 pandemic have focused on its impact on mental health professionals (Ornell *et al.*, 2020), and no systematic evaluation of the mental state of the elderly population has been made so far.

In spite of having a younger population compared to European countries, many structural deficiencies (or at least inequalities) in the Brazilian public health services across different regions of the country render the treatment of severe COVID-19 less effective, therefore increasing lethality rates. In addition, several social characteristics render the Brazilian population particularly prone to a high morbidity from the infectious disease, with a putative subsequent effect on mental distress. Brazil has the sixth largest population in world with over 211 million inhabitants (IBGE, 2020), and socio-demographic conditions may preclude physical distancing in densely populated communities. Certain cultural peculiarities also play an important role in the fast dissemination of the disease, with large and belonging families – frequently with two to three generations dwelling in the same household with limited private space and sanitation.

The first confirmed case of COVID-19 in Brazil was reported on 25th February 2020 in São Paulo, affecting a 61-year-old man who had just returned from Lombardy, the epicenter of the epidemic in Italy (MHB, 2020a). Three months later, São Paulo turned out to be the most affected state in Brazil, with almost 86,000 confirmed cases and 6,500 deaths from COVID-19, along with almost 400,000 confirmed cases and 25,000 deaths in the whole country, with mortality and lethality rates of 11.7% and 6.3% (estimates of 27th May) (SEADE, 2020). Currently, with the highest transmission rate (2.8) in an estimation involving 48 countries worldwide, casualty counts doubling every 5 days (*The Lancet*, 2020), the severe acute respiratory syndrome coronavirus 2 (SARS-CoV-2) health crisis in Brazil is heading straightforward toward the epidemic peak (MHB, 2020b). Brazil currently stands second in the world ranking of total number of cases and has become the epicenter of the pandemic in Latin America, with mortality numbers increasing steadily (WHO, 2020). The highest counts were so far registered in the most populated regions of south-eastern Brazil, particularly in the states of São Paulo and Rio de Janeiro. However, much less densely populated regions in the Amazon basin have also been severely affected. Many communities in these regions face important limitations in sanitation and access to health services, making the states of Amazonas and Pará (along with two other states in the north-eastern coast, i.e. Ceará and Pernambuco) the ones with highest mortality rates per 100,000 inhabitants (SEADE, 2020). The distribution of deaths across these states accounts for 81% of deaths reported in Brazil, and the percentage of people who have been infected with SARS-CoV-2 ranges from 3.3% in São Paulo to 10.6% in Amazonas (Mellan *et al.*, 2020).

### Greater vulnerability of older adults

The elderly represents one of the most vulnerable subgroups in the SARS-CoV-2 pandemic, with mortality rates in the 15–20% range according to Chinese estimates (Wu and McGoogan, 2020). Taking into account, infection/lethality rates of the disease in countries that have been affected at early stages of the pandemic, such as Italy and China, an international consortium generated a mathematical model to estimate the number of deaths from COVID-19 in Brazil in distinct age groups; the model projected that the highest proportion of deaths would occur among older adults (with 70 years of age or more), with casualty numbers varying from 120,000 to 720,000 depending on the infection rate (10% or 50%, respectively) that was used to feed the model (Martinez *et al.*, 2020). The greater vulnerability of the geriatric population is presumably accounted for by the fact that aging is associated with a reduction in the efficiency of immune response (Fried *et al.*, 2001) and more frequent comorbidities and chronic diseases such as hypertension, diabetes, cancer, and cardio-respiratory diseases (Landi *et al.*, 2020). Older adults are also more susceptible to emotional distress from different psychosocial predisposing factors (Valiengo *et al.*, 2016) which may be magnified in the COVID-19 crisis (Yang *et al.*, 2020). Distressing factors can be directly or indirectly related to the pandemic per se. In the first case, stress results from the fear of getting sick, and from the uncertain prognosis of the disease; from the limited availability of individual protection equipment; from the difficulties to get a proper diagnostic assessment in case of suspected infection; from the fear of not having access to adequate medical treatment; from the limited resources for the treatment of severe cases; and, ultimately, from grief and bereavement for those who have already lost a loved one. In the second case, mechanisms of stress may operate indirectly through cumulative losses that arise from sanitary measures and governmental acts, interfering in the daily living of many individuals – with social, economic, and cultural implications. A recent review suggested that individuals submitted to compulsory quarantine present with an array of symptoms of emotional distress, ranging from simple feelings of stress, fear, frustration, confusion, irritability, and anger to the actual onset of mood-related disorders, particularly, anxiety, depression, and sleep disorders (Asmundson and Taylor, 2020; Brooks *et al.*, 2020). Excessive coverage of the theme by the media – particularly in the light of conflicting information and recommendations – let alone the frequent and awkward conflicts between the various levels of the Brazilian authorities – may also exacerbate stress and uncertainties about the problem (Pfefferbaum and North, 2020). Furthermore, the fear of exposure to the risk of contamination, reinforced by compulsory quarantine and lockdown measures, may prevent older adults from attending follow-up medical appointments or going to emergency medical facilities in case of relapse of relevant (e.g. cardiovascular) comorbidities, leading to increase in all-cause mortality.

The new coronavirus pandemic has prompted some rapid and creative responses from mental health professionals in several affected countries. In Brazil, an important taskforce in this perspective was launched by the Department and Institute of Psychiatry HCFMUSP to provide psychological and psychiatric support for health care professionals working in the frontline of the pandemic fight (Miguel Filho *et al.*, 2020). All in all, there has been an important increase in the awareness of the contribution of mental health problems, as an important (although secondary) dimension to the preservation of global health as the COVID-19 crisis progresses worldwide. This movement has been observed both in the general population and in certain risk groups, such as among health professionals (Ornell *et al.*, 2020) and in older adults (Meng *et al.*, 2020; Qiu *et al.*, 2020). The latter are exposed not only to the risk of death from viral infection or its complications but also to complications of long-lasting emotional distress. Given the higher risk of psychiatric morbidity in older adults with pre-existing neuropsychiatric conditions, this specific population group requires well-established and specialized interventions to provide adequate clinical care (Lima *et al.*, 2020). The delivery of psychiatric care in such complex situations must also account for the individual necessities of older patients, in addition to a better knowledge about the risk and protection factors related to geriatric mental health. Such major challenges require the implementation of psychosocial interventions that must be delivered in real time and at large scale, requiring the incorporation of digitally based psychological support and psychiatric care to the exposed population, in addition of medically oriented therapies (Duan and Zhu, 2020; Xiao, 2020).

### COVID-19 and geriatric mental health: gaps to be filled

The negative impact of the COVID-19 crisis on geriatric mental health is unquestionable. However, there is limited information about the specificities of this impact on certain population groups, such as clinical (hospital-based) vs. community samples; urban vs. rural populations; or patients with distinct forms of pre-existing neuropsychiatric disorders. For instance, what is the pattern of response in sub-samples of patients with late-life psychotic disorders, or those with chronic mood disorders, such as major depression or bipolar disorder? What happens to older adults with neurocognitive disorders or intellectual disabilities, such as Alzheimer’s disease and Down syndrome? Therefore, there is urgent need do generate scientific evidence about the psychological and psychiatric needs of these individuals in the pandemic context, so that we can propose interventions to treat and/or prevent negative mental health outcomes. In this context, the LIM-27 Psychogeriatric Team at the Institute of Psychiatry HCFMUSP have recently launched a clinical initiative to screen for signs of emotional distress related to the COVID-19 crisis and to monitor the potential relapse of psychological and behavioral symptoms in patients with pre-existing neurocognitive and psychiatric disorders. The target sample (n = 500) comprises the total cohort of outpatients who attend two psychogeriatric clinics at IPq-HCFMUSP, a tertiary, university-based psychiatric hospital located in the largest public hospital complex in Latin America. This initiative combines clinical and scientific goals, namely the prospective assessment of the mental state of a population group at high risk for psychiatric morbidity in the light of the SARS-CoV-2 pandemic; the characterization of the emotional distress presented by psychogeriatric patients in the present pandemic context; and the identification of predisposing and triggering factors related to psychiatric morbidity in these individuals. Data will be captured by self-assessment questionnaires to be filled by the patient under the supervision of a qualified informant, and by clinician-assisted telephone interviews through which the Hospital Anxiety and Depression Scale (HAD) (Zigmond and Snaith, 1983) and the Neuropsychiatric Inventory-Questionnaire (NPI-Q) (Camozzato *et al.*, 2015) were administered. We intend to determine the prevalence of psychological and behavioral symptoms in a clinical sample of psychogeriatric patients, the impact of distressing factors, and their relation with the COVID-19 pandemic. Whenever necessary, we will undertake teleconsultations to perform an initial medical assessment and refer suspected cases to appropriate psychiatric care as needed.

Preliminary results from this study upon inclusion of the first 72 participants indicate that psychological distress in this population is frequent, with 37.7% of subjects reporting exacerbation of pre-existing symptoms and 20.8% reporting new, incident mental symptoms. Therefore, almost 60% of the assessed elders reported psychiatric or psychological distress related to present situation. Sleep complaints were reported by 57% of participants, including insomnia, inefficient sleep, or daytime sleepiness. The most frequent mood symptoms could be allocated into three clusters: anxiety (ranging from any physical and/or cognitive symptoms of anxiety to panic attacks); depression (from low mood, lack of interest and pessimism to severe forms); and dysphoric/mixed symptoms (e.g. irritability, anger, and elevated mood). Nonetheless, upon objective assessment, we found that the overall severity of reported symptoms was relatively low, as indicated by the output of psychometric scales. Mean scores on the anxiety and depression domains of HAD scale were 8.1 (SD 5.1) and 9.3 (SD 5.1), respectively, both indicative of “possible or questionable” diagnosis of an actual disorder. Neuropsychiatric symptoms, as assessed by the NPI-Q, were also relatively frequent, particularly among patients with neurocognitive disorders: depression/dysphoria were reported to be present in 64.3% of participants; anxiety in 50.0%; apathy in 65.7%; irritability in 60.0%; nocturnal behaviors in 67.1%, and appetite/eating behaviors in 58.6% – but again these symptoms were of mild-to-moderate severity, and caused minimal or mild distress to caregivers. Paranoid symptoms were reported by 23% of the participants, ranging from simple suspiciousness to exacerbation of delusional thoughts, the latter occurring among patients with pre-existing psychosis. Alcohol- and substance use-related problems were not an issue in this study group.

The present data, although preliminary, seem to be in accordance with reports from other studies conducted in the COVID-19 pandemic crisis. In a nationwide population study conducted in China (Qiu *et al.*, 2020), with over 50,000 individuals assessed by self-report questionnaires, older age (i.e. >60 years) was associated with higher estimates of psychological distress, which was supposedly reinforced by the higher morbi-mortalitly rates in this age group. Another epidemiological study conducted in China in a sample of 1,556 older adults showed that 37.1% of the participants had symptoms of anxiety or depression during the COVID-19 crisis (Meng *et al.*, 2020). The authors further suggested that, in addition to the higher prevalence of depression in this population and their limited access to mental health services, sanitary measures to restrain the dissemination of the virus, such as compulsory quarantine, may additionally intensify social isolation and feelings of solitude, therefore increasing depression rates. Such risk may be even higher among older adults with pre-existing psychiatric disorders, as our preliminary results suggest. Therefore, upon completion of the present study and through systematic data capture in a relatively large clinical sample, we intend to learn about the role played by the multiple risk factors related to mental distress and their relationship with the COVID-19 crisis. We intend to improve awareness about this matter in the Brazilian population, and reinforce adaptative, health-promoting behavioral changes in order to minimize the risk of getting mentally ill.

Experts and mental health professionals working in the pandemic context in eastern Asia in the previous months have reported many successful experiences, which strongly support the use of digital technologies and telemedicine to implement diagnostic and soft therapeutic interventions to deliver psychoeducation, psychological support and psychiatric counseling (Liu *et al.*, 2020; Yao *et al.*, 2020). We will also test the effectiveness of digital technologies to deliver diagnostic assessment, psychoeducation, psychological support and therapeutic counseling in the context of the COVID-19 crisis in Brazil. These methods are of course devoid of any risk of contamination through social contact, but nonetheless they need to be tested for safety and efficacy. In addition, these methods rely on the accessibility of the population to digital technologies, and on the availability of trained human resources to operate them. We still need to provide evidence of efficacy of these interventions on geriatric mental health care in different socioeconomic contexts. The level of evidence of efficacy for these interventions is still low, but there is room for initiatives that take into account the lessons that have been learnt from pioneer trials in Asia, provided we bear in mind the substantial variation in sociocultural aspects of studies conducted in different countries.
